# Finding antibodies in cryo-EM maps with CrAI

**DOI:** 10.1093/bioinformatics/btaf157

**Published:** 2025-04-09

**Authors:** Vincent Mallet, Chiara Rapisarda, Hervé Minoux, Maks Ovsjanikov

**Affiliations:** LIX, Ecole Polytechnique, IPP Paris, Palaiseau, 91120, France; Integrated Drug Discovery, Structural Biology and Biophysics, Sanofi, Vitry-sur-Seine, 94400, France; Digital R&D, Sanofi, Vitry-sur-Seine, 94400, France; LIX, Ecole Polytechnique, IPP Paris, Palaiseau, 91120, France

## Abstract

**Motivation:**

Therapeutic antibodies have emerged as a prominent class of new drugs due to their high specificity and their ability to bind to several protein targets. Once an initial antibody has been identified, its design and characteristics are refined using structural information, when it is available. Cryo-EM is currently the most effective method to obtain 3D structures. It relies on well-established methods to process raw data into a 3D map, which may, however, be noisy and contain artifacts. To fully interpret these maps the number, position, and structure of antibodies and other proteins present must be determined. Unfortunately, existing automated methods addressing this step have limited accuracy, require additional inputs and high-resolution maps, and exhibit long running times.

**Results:**

We propose the first fully automatic and efficient method dedicated to finding antibodies in cryo-EM maps: CrAI. This machine learning approach leverages the conserved structure of antibodies and a dedicated novel database that we built to solve this problem. Running a prediction takes only a few seconds, instead of hours, and requires nothing but the cryo-EM map, seamlessly integrating within automated analysis pipelines. Our method can find the location and pose of both Fabs and VHHs at resolutions up to 10 Å and is significantly more reliable than existing approaches.

**Availability and implementation:**

We make our method available both in open source github.com/Sanofi-Public/crai and as a ChimeraX bundle (crai).

## Graphical Abstract

**Graphical Abstract:**
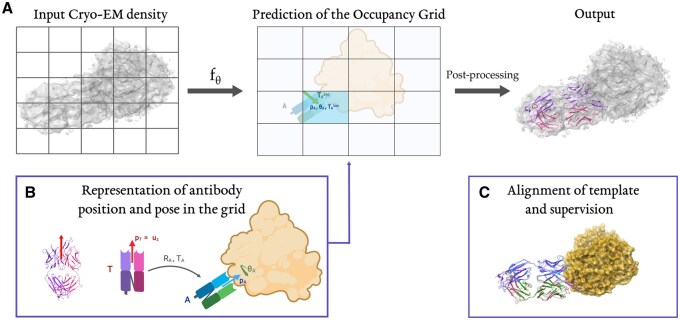

## 1 Introduction

Since the first monoclonal antibody entered the clinic in 1986 (Emmons and Hunsicker 1987), antibody-based therapeutics have made considerable progress. With over a hundred compounds approved and forty just in the last three years (Kaplon *et al.* 2023, TheAntibodySociety 2023), the use of antibodies currently appears as one of the most promising approaches for designing new treatments for patients. Antibody-based therapeutics rely on the identification of antibodies that can bind to a target molecule (antigen) with high specificity through their tips called the Complementarity Determining Regions (CDRs). Monoclonal antibodies (mAb) are the most widely used antibodies. They are composed of two domains called the Antigen Binding Fragment (Fab) and a central stalk that binds to the Fc receptor. More recently, antibody fragments consisting of a single variable antibody domain, engineered from heavy chain antibodies generated by camelids (Arbabi Ghahroudi *et al.* 1997) (nAbs or VHHs) have attracted interest as an alternative to mAbs (Duggan 2018, Jovčevska and Muyldermans 2020). Whether for mAbs or VHHs, initial hits are typically found using immunization (Köhler and Milstein 1975, Jones *et al.* 1986) or phage display (Smith 1985, McCafferty *et al.* 1990). Before entering clinical studies, initial hits need to be optimized with regard to several properties including their efficacy, manufacturability and safety. Among those properties, specific binding to the antigen is a critical objective scrutinized from the early phases of the process until drug candidate selection. Binding optimization relies on obtaining the structure of the initial hit since the knowledge of the atomic coordinates at the contact points between the Ab and its target improves the understanding of its mode of action and guides the optimization of the binding affinity (Chiu *et al.* 2019).

Cryogenic Electron Microscopy (cryo-EM) has become the most common way to experimentally obtain protein structures of therapeutic antibodies bound to their target. In cryo-EM, the target protein is embedded in ice and exposed to an electron beam, resulting in raw noisy images of individual particles. These raw 2D images are then aligned and transformed into a 3D Coulomb potential map (Scheres 2012, Punjani *et al.* 2017) from which atomic coordinates are inferred. Recent advances in data collection hardware and software, along with improved data processing pipelines, have increased data output. As a result, more academic labs and global pharmaceutical industries have adopted the technology (Peplow 2017). Recently, a new data collection workflow has been shown to produce 3–4 Å structures of a pharmaceutically relevant target protein with 1 h of instrument time, thus allowing the theoretical resolution of 24 structures a day (Cushing *et al.* 2023). However, this raw data needs to be processed from micrographs to molecular structures. While data collection is rapid, current data *processing pipelines* rely on significant manual intervention for simple tasks and decisions, and ultimately take days and weeks to complete.

The recent rise of artificial intelligence-based methods for protein structure prediction holds promise but cannot yet accurately model the interaction between antibodies and their epitope. Current methods fail to accurately model the diverse CDR loops that are critical for antigen binding (Hummer *et al.* 2022) calling for significantly more structural data. Unfortunately, the reliance of existing methods on manual intervention significantly hinders cryo-EM map analysis at scale, required for such understanding.

As automation has allowed X-ray crystallography to become a key technique in the structure-based drug discovery pipeline (Blundell and Patel 2004), so it should for cryo-EM, making it cheaper and faster, and freeing the time of researchers from button-pressing tasks, to structure interpretation and drug engineering. The process of automation is being accelerated by the application of machine learning to the different stages of the pipeline, from data acquisition (Bouvette *et al.* 2022, Fan *et al.* 2022), to preprocessing of micrographs (Sanchez-Garcia *et al.* 2020), particle picking (Wang *et al.* 2016, Bepler *et al.* 2019, Wagner *et al.* 2019, Zhang *et al.* 2019, Yao *et al.* 2020), 2D class selection (Kimanius *et al.* 2021), 3D heterogeneity deconvolution (Matsumoto *et al.* 2021, Zhong *et al.* 2021), and analysis (Pearce *et al.* 2017, Brzezinski *et al.* 2021, Karolczak *et al.* 2024).

Unfortunately, the last step of attribution of the map (fitting of atomic coordinates of a protein into the map), remains a tedious analysis bottleneck. It is still a largely manual process typically done using ChimeraX (Pettersen *et al.* 2004), followed by the local optimization of atomic coordinates using Coot (Emsley *et al.* 2010). Some techniques have been developed to help automate this process (Liebschner *et al.* 2019), although with limited accuracy. Furthermore, the methodological challenges associated with this problem have so far impeded the automation of this step using existing machine learning approaches. Specifically, the data is noisy and heterogeneous and the output is high dimensional, making off-the-shelf computer vision methods irrelevant. Tools based on machine learning to trace the sequence in the map were recently developed with good results (Pfab *et al.* 2021, Jamali *et al.* 2024, Wang *et al.* 2024). They are, however, limited to resolutions better than 4 Å and can exhibit prohibitive running times.

In the context of using cryo-EM for optimization of therapeutic Abs, we aim to address the problem of finding Abs (Fabs and VHHs) in cryo-EM maps. To achieve this, we propose CrAI, the first fully automatic and efficient machine learning based approach that is applicable at all resolutions better than 10 Å without any additional inputs beyond the map. To develop our solution, we introduce a customized deep learning technique, which takes into account and exploits the structural properties of this problem setting. In particular, we leverage the conserved structure of Abs as a prior information (Cohen *et al.* 2022) to formulate our problem as a special instance of 3D object detection (Qi *et al.* 2019, Zou *et al.* 2023). We gather a novel database of aligned Ab structures and Cryo-EM maps, and use it to train a model with a custom loss that involves optimal transport supervision. We test our tool on a set of 215 maps of various resolutions, containing 374 Fabs and 86 VHHs. We successfully find Abs in over 90% of systems, outperforming existing methods by a margin of 25%, while exhibiting *one thousand times* speedup. We make our tool available as a ChimeraX bundle to facilitate adoption.

## 2 Materials and methods

### 2.1 Building a database

We build a curated antibody database to train and test our method, comprising Fabs and VHHs. Fabs are composed of one constant and one variable domain for each of the heavy and the light chains. The two variable domains are denoted as the Fv variable fragment (Fv). VHHs are antibody fragments that correspond to the sole variable domain of the heavy chain. They represent a promising family for therapeutic antibodies.

The Fab data are originally fetched from *SabDab* (Dunbar *et al.* 2014) in the form of a list of protein chains. We fix a few broken annotations, notably for systems containing both Fabs and VHHs (more details in Supplementary Section S1.2). Using the PDB (Berman *et al.* 2000), we find the corresponding cryo-EM maps and download all corresponding maps and structures. Finally, we remove systems with resolution below 10 Å or ones with no antibodies or antigen chains, yielding a total of 1032 maps. The maps include both Fabs and Fvs, but the constant region of Fabs is often missing in the deposited structure. To avoid false negatives in our data, we chose to always predict the position of the Fv as this is the only part consistently reported.

The resulting map files can be enormous (up to 10^9^ grid cells) especially for symmetric assemblies, such as viruses, where the asymmetric unit only occupies a fraction of the map (e.g. pdb 7kcr). Since some regions of the map correspond to proteins omitted in the deposited structure, using whole maps would create artifacts of negative labels. To limit those artifacts in our dataset, we crop the original maps around the structure with a margin of 25 Å. Additionally, we resample maps to a fixed voxel size of 2 Å and normalize them by zeroing out negative values and dividing by the maximum of the map.

We split this dataset following a temporal splitting strategy with more recent systems in the test split (denoted as sorted setting). While this procedure is often used to provide a realistic use case, it can also introduce a bias, for instance toward structures of the spike protein of SARS-Cov-2 obtained during the COVID pandemic. Hence, we also report our performance following a random split (random setting). Given the stable performance across these splits and the high computational cost of training, we use a single random split. We obtain 722, 155, and 155 systems in the train, validation, and test splits, respectively. Note that a single system can include several Fabs. On average, there are 2.25 Fabs per system in our dataset. The number of Fabs per split is 1627, 320, and 374, respectively—with similar numbers for the random split. We now have a split database of cropped, resampled, normalized cryo-EM maps containing Fabs.

All of these steps are repeated to create a VHH database. We started from *SAbDab-nano* (Schneider *et al.* 2022), applied the same filters on the raw data, resulting in 398 systems, and performed the random and sorted splitting procedures as above. This amounts to 278, 60, 60 systems in each split containing 458, 74, 86 VHHs for a mean of 1.55 VHHs per system.

### 2.2 Overview and motivation

**Figure 1.**
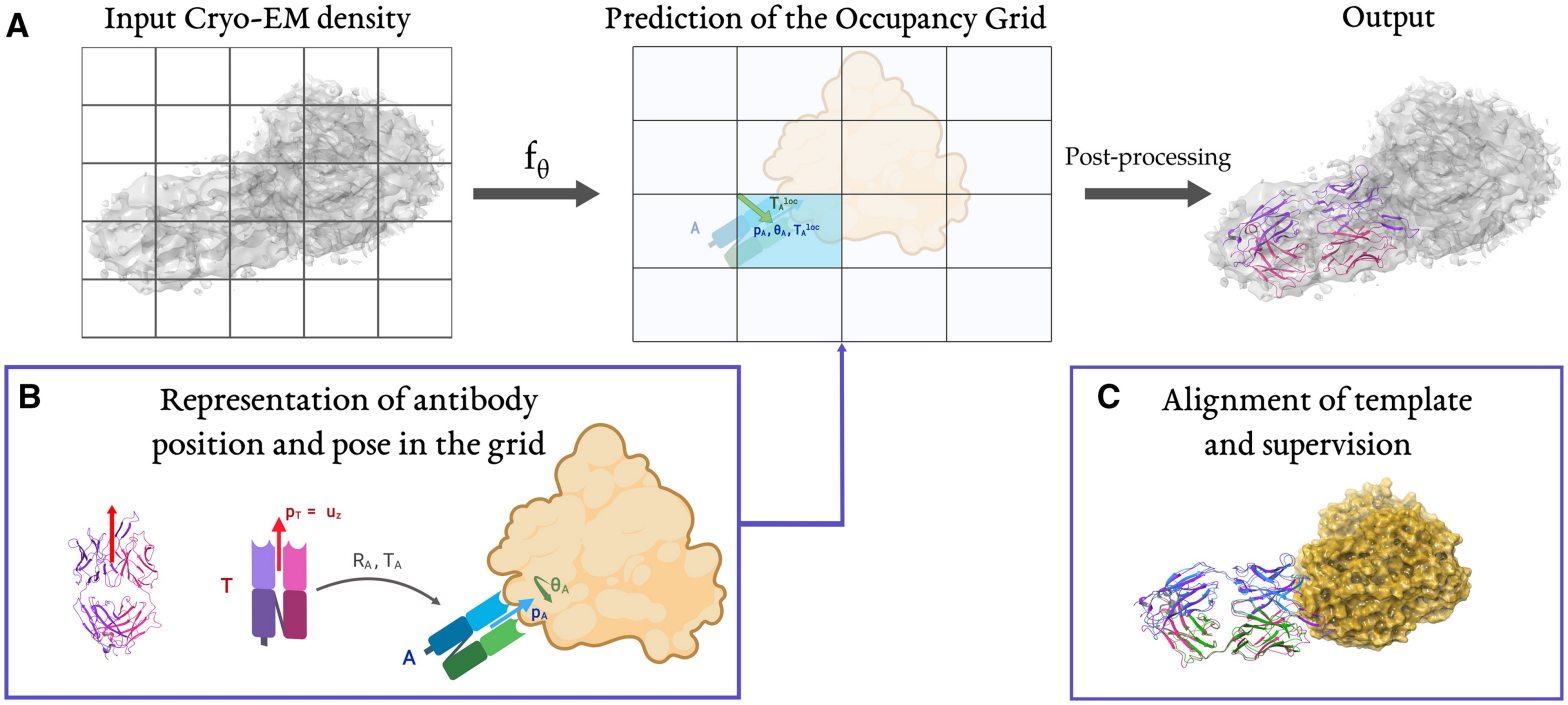
(A) CrAI predicts an occupancy grid that represents antibodies found in an input map. The prediction of this occupancy grid can be post-processed into a PDB containing the predicted antibodies’ structures. (B) The atomic structure of our template (pdb 7lo8) is displayed next to its cartoon, with ${\vec{u}}_{z}$ shown in red. We compute optimal alignments $R_{A}^{*},T_{A}^{*}$ of our template onto Abs. We decompose the rotation into $R_{A}^{*}=({\vec{p}}_{A},\theta_{A}$) and the translations into a position in a grid $T_{A}^{\mathrm{cell}}$ and an offset from the grid corner $T_{A}^{\mathrm{loc}}$. We thus obtain a grid with zero values except for cells containing an Ab, in those cells, we have ${\vec{p}}_{A},\;\theta_{A}$ and ${\vec{p}}_{A},\;\theta_{A}$. (C) Example alignment of our template (*red, purple*) with the experimental structure of system 6bf9: antigen (*orange*) and Fab (*blue, green*). As can be seen, our template aligns well to other antibodies (RMSD = 1.8 Å).

CrAI detects antibodies in cryo-EM maps using a customized deep learning-based technique, trained on our curated dataset comprised of 1430 cryo-EM maps containing Fabs and VHHs. When designing the model, we introduce a custom representation of the structure of antibodies to facilitate the learning process (Fig. 1B) and train a neural network to predict this representation. Once the model makes a prediction, persistence diagrams (Carlsson *et al.* 2004, Carlsson 2009) are used to select the relevant results which are transformed into a PDB file. Remarkably, at prediction time, this procedure does not require anything beyond the input Cryo-EM map. Our pipeline is illustrated in Fig. 1.

The design of our approach, CrAI, is motivated by several methodological challenges. First, due to the challenges inherent in the limited scale and significant noise present in the training data, we aim to incorporate prior information to make learning more data-efficient and robust. Specifically, we leverage the *conserved nature* of antibodies to approximate the detailed structure of the output by its *position* and *orientation*. Moreover, we use a custom parametrization of the rotation that takes into account a biologically *expected* pose, while being flexible to allow arbitrary orientations. Finally, the list of such representations of antibodies for a system is transformed into a grid overlaid over the cryo-EM map, such that the position of an antibody is encoded as an offset from a grid cell. The encoding of the output is shown in Fig. 1B.

Second, we introduce a fully convolutional design to accommodate arbitrary grid sizes that might be present at inference time. We also train the network with rotation augmentation to approximate rotation equivariance and accommodate arbitrary orientations.

Finally, we introduce a custom training loss that incorporates a formulation based on Optimal Transport along with persistence diagrams to better capture the geometric aspects of our problem, such as predicting nonoverlapping objects and including *distance*-based penalties between our prediction and the ground truth.

### 2.3 Problem formulation

Starting from an input cryo-EM map, we want our method to output the 3D coordinates of one or several Abs. Because of the highly conserved structure of Abs, we simplify our problem by only predicting how to align a fixed antibody template **T** (pdb 8fab) with the Abs, without deformations. We computed the optimal alignments with pymol align (DeLano 2002) during data pre-processing. We provide an example alignment of our template in Fig. 1A.

More formally: Let X be the cryo-EM map we consider, $n_{X}$ the number of Abs contained in this map and $A_{X}=\{A_{i},0\leq i<n_{X}\}$ the set of such Abs. Given an Ab **A** and a registration objective d, let $R_{A}^{*},T_{A}^{*}=\arg\;\min_{R,T\in (SO_{3}(R)\times R^{3})}d(A,RT+T)$ be the translation and rotation that best align **T** to **A**. Finally let $S_{X}=\{(R_{A_{i}}^{*},T_{A_{i}}^{*}),A_{i}\in A_{X}\}$ be the set of optimal alignments. Note $S_{X}$ consists of elements of the Euclidean group in 3D that is 6D, whereas elements of $A_{X}$ are 3D coordinates for hundreds of atoms. In this article, we aim to predict $S_{X}$ instead of $A_{X}$.

The optimal rotations mentioned above can be parameterized in many ways. Let ${\vec{p}}_{A}$ denote the unit vector oriented from the center of mass of an Ab A toward its antigen. Since canonical binding tends to happen through the CDRs, we observe that it is easier to predict ${\vec{p}}_{A}$ than the rotation around ${\vec{p}}_{A}$. Therefore, we decomposed $R_{A}^{*}$ into a rotation transforming $R_{A}^{*}$ into ${\vec{p}}_{A}$ and one 2D rotation around ${\vec{p}}_{T}$ of angle $\theta_{A}$. The generality of this decomposition is established in Supplementary Section S1.1 and its relevance is shown in Fig. 3B.

Our problem is now formulated as an object detection problem, which consists of detecting, localizing, and aligning the Abs in a given map. Following common practice in the object detection literature (Redmon *et al.* 2016), we overlay an occupancy grid $G^{X,S}$ of size **S** over our input: this grid contains ones for cells encompassing an Ab and zeros elsewhere. The size of the cells of this grid corresponds to a fixed spatial volume and does not depend on the resolution of the map. We then decompose each translation into two parts: one going to the corner of the occupied cell and one from this corner to the Ab: $T_{A}^{*}=T_{A}^{\mathrm{cell}}+T_{A}^{\mathrm{loc}}$, as shown in Fig. 1A. Hence, finding the optimal translation comprises finding occupied cells and local translations in those cells. This decomposition makes the encoding of the output invariant to translation and avoids manipulating large values to encode translations in the grid.

### 2.4 Architecture and learning procedure

We aim to solve the object detection problem stated above with a Machine Learning approach, trained on our dataset. Given a cryo-EM map of size *S_i_*, $X\in R^{S_{i}}$ and its corresponding occupancy grid of size **S**, $G^{X,S}$, our network is a function $f_{\theta }:R^{S_{i}}\overset{}{\to }R^{10\times S}$ such that $\hat{y}=f_{\theta }(X)\in R^{10\times S}$ is our prediction for X. The prediction at a position *s* is denoted as ${\hat{y}}^{k}(s)\in R^{10},$ where k=1..10. The first dimension of this output, ${\hat{y}}^{0}(.)$, is a prediction of the occupancy grid $G^{X,S}$. The nine other dimensions are predictions in each cell relative to the putative Ab contained in it. The details about both the exact role of each dimension as well as our training procedure are described below.

The architecture of our model $f_{\theta }$ is a 3D UNet (Çiçek *et al.* 2016) with a depth of 4. To enhance robustness, the network is trained using data augmentation, with the eight possible rotations over a grid and random cropping inside the input grid up to three cells. We used Adam optimizer over 1000 epochs. Hyper-parameters and exact architecture were not extensively tuned to avoid artificially boosting performance. Finer details about the ones we used can be found in Supplementary Section S1.3.

### 2.5 Custom loss

#### 2.5.1 Prediction of the right cells using optimal transport

For convenience, in this section, we drop indices and denote the ground truth occupancy grid $G^{X,S}$ as G. The first slice of our output $\hat{y}$ is a prediction of the occupancy grid and hence, let us denote it as ${\hat{G}}_{s}={\hat{y}}^{0}(s)$. In order to make $\hat{G}$ close to G, we will use two loss terms.

Because most grid cells are unoccupied, our prediction is very imbalanced. Hence, our first loss term is a weighted binary focal loss (Lin *et al.* 2017) that focuses on cells with *wrong* predictions:

$$\mathrm{foca}l_{\gamma ,\lambda }(\hat{y},y)=\lambda y{\left(1-\hat{y}\right)}^{\gamma }\;\text{log}(\hat{y})+(1-y){\hat{y}}^{\gamma }\;\text{log}(1-\hat{y}),$$

 

$$L_{1}(\hat{G},G)=\sum_{s\leq S}\mathrm{foca}l_{\gamma ,\lambda }({\hat{G}}_{s},G_{s}).$$

We observe that this focal loss does not consider the *distance* between our prediction and the ground truth: predicting the neighbor pixel results in the same loss value as predicting the opposite side of the map. To address this issue, we add an *optimal transport* term to our loss, denoted as $L_{2}$. This term gives meaningful supervision to all voxels of our grids and depends on the distance to the closest occupied voxels.

After normalization, we can view $\hat{G}$ and G as measures defined over the regular grid. $L_{2}$ is a regularized, corrected version of optimal transport called Sinkhorn divergence applied to our normalized predictions and targets. We computed this term with the *GeomLoss* (Feydy *et al.* 2019). We refer to Supplementary Appendix SA.4 and to Peyré and Cuturi (2019) and Feydy *et al.* (2019) for a more detailed discussion of the computation. The relevance of this loss is assessed in the Results section.

#### 2.5.2 Prediction within each cell

Let **A** be an antibody in our system and $s_{A}$ its position in the grid. Beyond predicting the right grid cell, we also want finer grained prediction about its precise position in the cell $T_{A}^{\mathrm{loc}}$, its orientation $\left({\vec{p}}_{A},\theta_{A}\right)$ and its classification as a Fab or a VHH. Let ${\hat{y}}_{A}^{j}={\hat{y}}^{j}(s_{A})\in R^{10}$ be the prediction at this position, we will introduce additional loss terms to capture these finer grained predictions. We emphasize that these will only be applied on grid cells containing an antibody.

To predict the right pose of **A** in the cell, we use three values to predict the offset from the corner of the grid cell, $T_{A}^{\mathrm{loc}}$ learnt with a mean squared error loss $L_{3}$. The following three values are used to predict ${\vec{p}}_{A}$ by directly predicting its coordinates. The corresponding loss, $L_{4}$, is composed of a dot product term to control the direction of the prediction along with a term to make this vector unit norm. Favoring unit norms avoids numerically unstable normalizations. The remaining two values are used to predict the angle $\theta_{A}$. Instead of directly predicting the angle, we aim to predict $u_{A}\in R^{2}$, the vector of polar coordinates $\left(1,\theta_{A}\right)$. This formulation avoids singularities and was shown to be beneficial when predicting angles (Jumper *et al.* 2021). Hence, $L_{5}$ has a similar form than $L_{4}$ in two dimensions to predict $u_{A}$. Using the notation ${\hat{y}}_{A}^{j:j+k-1}$ to denote the k dimensional vector obtained from the concatenation of ${\hat{y}}_{A}^{j},{\hat{y}}_{A}^{j+1}\cdots {\hat{y}}_{A}^{j+k-1}$, we end up with the following losses:

$$L_{3}({\hat{y}}_{A}^{1:3},T_{A}^{\mathrm{loc}})=\mathrm{mse}({\hat{y}}_{A}^{1:3},T_{A}^{\mathrm{loc}}),$$

 

$$L_{4}({\hat{y}}_{A}^{4:6},p_{A})=1-\langle {\hat{y}}_{A}^{4:6},p_{A}\rangle +\mathrm{mse}(||{\hat{y}}_{A}^{4:6}||,1),$$

 

$$L_{5}({\hat{y}}_{A}^{7:8},u_{A})=1-\langle {\hat{y}}_{A}^{7:8},u_{A}\rangle +\mathrm{mse}(||{\hat{y}}_{A}^{7:8}||,1).$$

Finally, as we use a single model for both Fabs and VHHs, we have a term that represents the probability that the object contained in the grid cell is a VHH and not a Fab. Let $\delta_{n}(x)$ be the indicator function for VHHs (one if VHH else zero). We again construct a weighted focal loss, with a weight of $\lambda_{n}=1000/400$ corresponding to the ratio of VHHs to Fabs, yielding the last loss,

$$L_{6}({\hat{y}}_{A}^{9},A)=\mathrm{foca}l_{\gamma ,\lambda_{n}}({\hat{y}}_{A}^{9},\delta_{n}(A).$$

To train our network we use a weighted sum of previous loss terms as the final loss. We sum the first two global ones and the sum of the four others over each antibody in our system:

$$L_{\mathrm{tot}}(\hat{y},X)=\sum_{i=1}^{2}L_{i}(\hat{G},G)+\lambda_{s}*\sum_{i=3}^{6}\sum_{A\in A_{X}}L_{i}(\hat{y_{A}},A).$$

We use values of 4, 30, and 0.2 for γ,λ, and *λ_s_*, respectively, without extensive tuning, as they were empirically found to give good results.

### 2.6 Post processing

A well-known problem with object detection is the possibility that the network predicts overlapping objects. Given the size of occupancy grid cells (around 8 Å), adjacent cells could not both contain the center of mass of a Fab. Hence, high values for adjacent cells typically amount to the detection of the same underlying object. Non Maximal Suppression (NMS) algorithms are used to discard such redundant predictions. Starting with our grid ${\hat{y}}_{0}$, we want to obtain a list of the distinct local minima. In this paper, we used an approach based on *Persistence Diagrams* (PD), implemented with *cripser* (Chazal *et al.* 2013, Tralie *et al.* 2018, Kaji *et al.* 2020). Simply put, we decrease a threshold probability value from the maximum value of our grid ${\hat{y}}_{0}$ and keep track of cells above this threshold. When the value of a cell goes over the threshold, either it has no neighbors in a visited connected component, giving *birth* to a new one, or all neighboring components are merged into the one with lowest initial values and others *die*. The difference between the values of death and birth are called lifetimes. We return connected components sorted by lifetimes.

This procedure takes into account both the *value* of a minimum and its *location* with respect to other minima. If we suppose that the number of objects to find is known, we keep proceeding until this number is reached (and refer to this setting as num). Otherwise we retain all predictions above a lifetime threshold. We use a threshold of 0.2 that was found to work best on the validation set (see Supplementary Section S1.5). We will denote this setting thresh. Interestingly, this procedure allows us to automatically detect the numbers of Abs in a map, without any prior information.

From our input map, we now have a list of predicted values. For each of those, we choose a template based on the classification in Fabs and VHHs and move this template to the predicted location and pose. We save the result as a file in PDB format.

## 3 Results

### 3.1 Baselines and metrics

Independent models were trained on the training sets obtained with the random and sorted splits (see Methods Section). We make inference of those models on their respective test sets, providing the network with the number of Abs to find (denoted as num) or relying on automatic thresholding to infer this number (thresh). For each system, a prediction method results in one or several predicted Ab positions. We report our results for individual Abs (ab) as well as aggregated by systems (sys) so that a system with many Fabs does not influence the results significantly.

When evaluating different approaches, individual predicted antibody positions need to be matched with actual antibody positions. This matching process is accomplished using the Hungarian algorithm (Kuhn 1955) on the distance between the center of mass of predicted and actual antibodies.

To the best of our knowledge, no tool enables predicting the position of antibodies solely from a Cryo-EM map. We benchmark against dock_in_map (Liebschner *et al.* 2019), a tool that takes the map along with the known atomic protein structures that need to be docked in the map. Considering that at test time, one does not have access to the ground truth structure, we ran dock_in_map with a fixed template Fab or Fv. Additionally, we ran dock_in_map with the actual structure giving us an upper bound of its performance.

The distribution of distances between centers of mass of the predictions and experimental structures shows that we outperform dock_in_map, even in the idealized scenario that uses the ground truth structure (see Supplementary Fig. SA.2). In the following, we will compare to this idealized and more challenging scenario as our baseline. Moreover, we observe that the predicted distances are bimodal: a first peak corresponds to systems predicted successfully and another spread mode corresponds to failed prediction.

We thus define a prediction as a true positive if it is closer than 10 Å to the ground truth. Performance is not very sensitive to the choice of this threshold as can be seen in the histogram. Undetected systems are false negatives and failed predictions are false positives. Our method’s ability to detect antibodies is assessed using F1 scores and reported in Table 1. This metric matches recall and precision for the baseline and the num settings since in those scenarios, any missed prediction (false negative) also results in an extra prediction (false positive). Recall and precision in the thresh setting are reported in Supplementary Table SA.1 and lead to a consistent analysis.

**Table 1.** Detection performance of the benchmark tool dock_in_map and of CrAI.^a^

	random Split				sorted Split				Mean			
	F1 (sys)	F1 (ab)	Distance	RMSD	F1 (sys)	F1 (ab)	Distance	RMSD	F1 (sys)	F1 (ab)	Distance	RMSD
Performance on Fabs												
dock_in_map	66.6	61.9	**0.78**	**1.87**	71.3	66.0	**0.68**	**1.69**	69.0	64.0	**0.73**	**1.78**
CrAI num	97.3	96.7	1.40	6.21	**96.9**	**95.7**	2.06	6.07	**97.1**	96.2	1.73	6.14
CrAI thresh	**98.1**	**97.6**	1.39	6.36	96.1	**95.7**	2.03	5.83	**97.1**	**96.7**	1.71	6.10
CrAI num + FitMap	93.1	91.2	1.12	5.41	93.8	92.0	0.83	4.12	93.5	91.6	0.98	4.77
CrAI thresh + FitMap	93.8	91.9	1.15	5.44	93.6	92.4	1.15	3.79	93.7	92.2	1.15	4.62
Performance on VHHs												
dock_in_map	77.8	68.8	**0.29**	**0.80**	90.5	89.1	**0.76**	**1.85**	84.2	79.0	**0.53**	**1.33**
CrAI num	**100.0**	**100.0**	0.99	4.22	**90.9**	**90.6**	2.32	7.59	**95.5**	**95.3**	1.66	5.91
CrAI thresh	99.4	99.3	1.01	4.18	90.3	88.4	2.14	7.07	94.9	93.9	1.58	5.63
CrAI num + FitMap	92.0	84.4	1.13	3.78	89.2	89.1	0.83	5.85	90.6	86.8	0.98	4.82
CrAI thresh + FitMap	91.4	83.7	1.00	3.66	88.6	86.8	1.28	5.40	90.0	85.3	1.14	4.53

a We either provide the ground truth number of objects (num) or not (thresh), and optionally post process our results with FitMap. We report the F1 score on different data splits (random or sorted), aggregated by systems (sys), or not (ab). These include 155 systems containing 374 Fabs and 60 systems containing 86 VHHs. We additionally report the mean distance and RMSD of successful predictions. Best value in bold.

### 3.2 Antibody detection performance

*CrAI accurately finds Abs.* Regarding the prediction of Fabs, in the first two rows, note that CrAI drastically outperforms dock_in_map in terms of F1. This holds true in all settings for an overall F1 going from approximately 69%–97%.

The prediction of VHHs is more challenging because we have less training data (278 instead of 722 training examples), they display less canonical binding modes and are smaller and tend to be more buried into the map—as opposed to Fabs often sticking out. Despite those challenges, we maintain our performance on VHH, losing only three F1 score points, as can be seen in the top rows of the bottom part of the table.

*CrAI can be used with an unknown number of Fabs.* We now evaluate CrAI in the scenario where it also automatically estimates the *number* of Abs (num), and thus the network is given *only the map* without any additional information. This is to compare to the result of dock_in_map that is additionally given the *experimental structures of all antibodies* that are to be found in the map. As shown in the second and third lines of Table 1, we retain most of the performance even in this more challenging, and more realistic setting. We find this remarkable, since the maps are highly heterogeneous and contain between one and six Abs and sets our method apart from *any* existing method in terms of its ability to detect antibodies in Cryo-EM maps in a fully automatic manner.

*CrAI runs fast, at all resolutions.* The average runtime of dock_in_map on our validation set is a prohibitive 883 s/system, which does not account for a few systems that we stopped after 5 h of computations. In comparison, our tool runs in *0.47 s/system*, i.e. more than a thousand times faster when using a GPU (A40). Even when using only one CPU, our tool runs in 1.9 s/system, four hundred times faster than dock_in_map and fast enough for this computational step to integrate seamlessly in an analysis pipeline. This stems from the complexity of our algorithm, that is linear with regards to the grid size.

**Figure 2.**
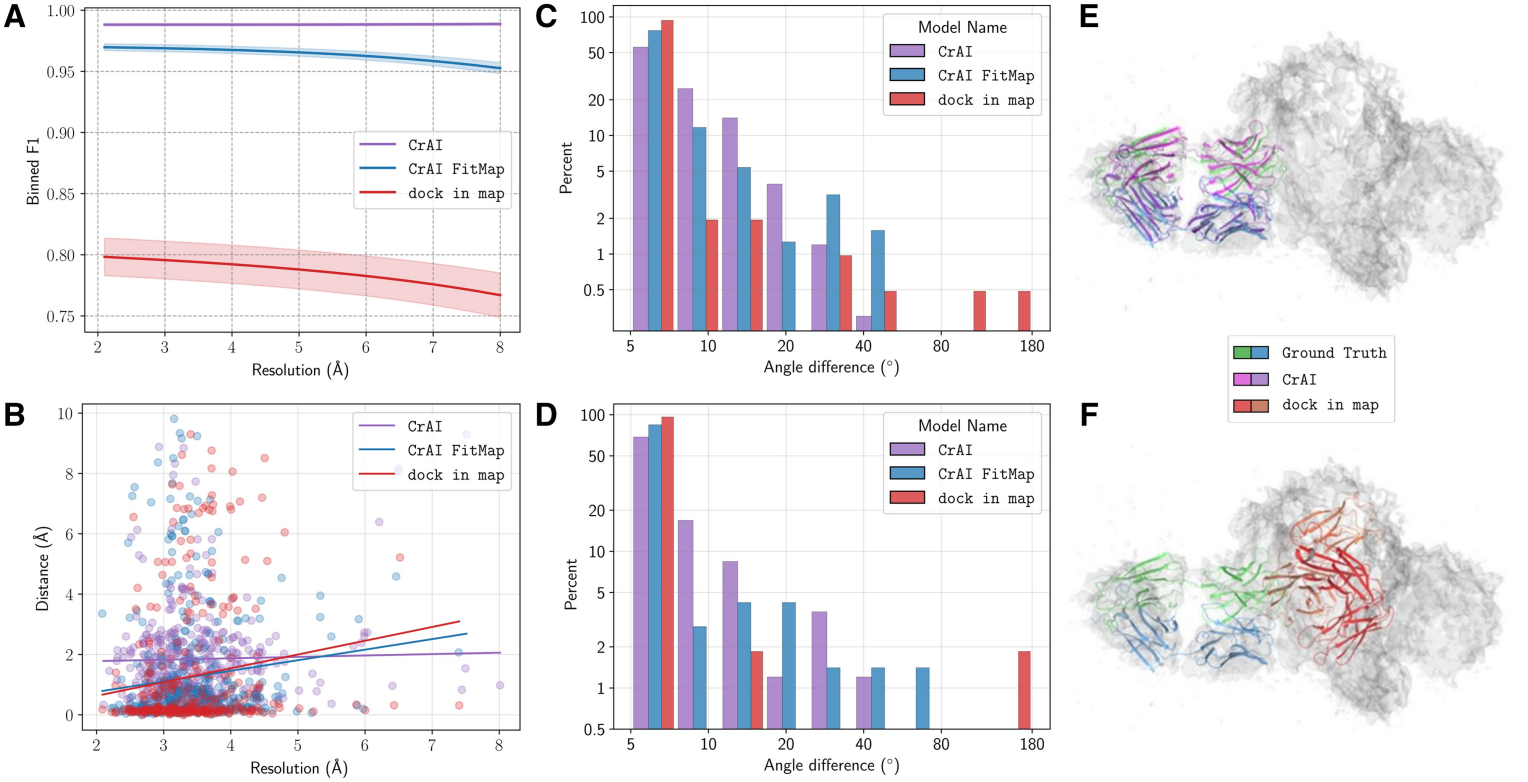
In (A, B, C, and D), we compare the performance of our CrAI thresh model, optionally post-processed with FitMap and of dock_in_map. We report the binned F1 score (A) and the distance of successful prediction (B) as a function of the resolution of the systems. We also report the distribution of the angle between the predicted and experimental ${\vec{p}}_{A}$ vector for Fabs (C) and VHHs (D). In the right column, we show the superimposition of the Fab from the PDB structure 7oh1 (blue/green) with the CrAI results (*purple*) (see E) and the dock_in_map results (*red*) (see F).

Figure 2A and B shows how our performance depends on resolution of the input map, over all of our datasets. Contrary to dock_in_map, we do not see a correlation and thus CrAI is *robust to low resolution*. This is a novel result as machine learning methods for tracing such as ModelAngelo (Jamali *et al.* 2024) only work for resolutions better than 4 Å.

*CrAI predicts correct positions.* On successful predictions, the center of mass of the prediction is closer than 2 Å to the one of the ground truth, which is close to optimal considering the map resolutions. The observed RMSD are around 6 Å. Given a successful prediction, dock_in_map appears to be a bit more precise. However, this is expected as dock_in_map uses the ground truth structure instead of a template, which has a 1.6 Å RMSD to the ground truth on average. Moreover, distances are computed only over successful systems and thus include *20% more systems in the CrAI column*.

To make our predicted positions more precise, one can first rapidly screen a map with a high F1 using CrAI, then precisely refine results in a local region using ChimeraX fast local refinement tool, FitMap. This additional step takes 0.4 s/system. It allows our prediction to have better distances and RMSD (4 Å), but slightly decreases performance and robustness to low resolution. This possibility is enabled in the proposed ChimeraX bundle.

*CrAI finds meaningful poses.* After validating the position of our predictions, we consider the predicted poses: are Abs in the correct orientation? Using the decomposition of rotations into predicting a vector ${\vec{p}}_{A}$ and an angle $\theta_{A}$, we can compute the angles between predicted values and actual ones. We provide histograms for the distribution of these angles in Fig. 2C and D, to show that most systems are predicted accurately. For the Fab data, the angle between ${\vec{p}}_{A}$ and its prediction is on average of $7.8^{{}^{\circ}}$ and the one for $\theta_{A}$ is $11.0^{{}^{\circ}}$, low enough values to make the prediction almost overlap with ground truth. For the VHH data, those values are on average $7.5^{{}^{\circ}}$ and $9.7^{{}^{\circ}}$, respectively. dock_in_map predicts more accurate orientations, which again stems from the use of the ground truth structure instead of a template. Post-processing with FitMap increases the pose accuracy on successful predictions but decreases it for low quality systems.

### 3.3 Further analysis

*Validation on true negatives.* We gather apo systems, using UniProt codes of antigen chains in our sorted test set, fetching other PDBs containing those codes and filtering them to not be included in SabDab or SabDab-nano, and to not be a virus, resulting in 26 apo systems.

Although CrAI predicted that 12 systems contain an antibody, we found 8 of these systems actually included an antibody that was missing in SabDab, and have contacted the authors to include them in later releases (details in Supplementary Section S2.2). This results in a precision of 77% on empty maps. Some erroneous predictions occur when artifacts corresponding to small values occur in the map, and are not automatically discarded. Providing a reasonable threshold enhances the precision to 89%. We make this option available in the plugin.

*Case studies.* To provide a visual example of the prediction of our tool, we picked a low-resolution system containing a Fab (pdb 7oh1). This system represents the neurotoxin LC-HN domain in complex with TT110-Fab1 at a resolution of 8.0 Å (Pirazzini *et al.* 2021). After a failed crystallization attempt, authors obtained a cryo-EM structure that enabled them to identify the mechanism of action of a neutralizing antibody against viral infection with tetanus. The suggested Fab represents a promising lead for prophylactic and therapeutic use. We found out that CrAI correctly positions the Fab but dock_in_map misplaced it in the core of the antigen (see Fig. 2E and F).

We provide an extended analysis on five additional systems of the random split test set in Supplementary Section S2.3, chosen because of their relevance in the context of drug discovery. Three systems contain Fabs and two include VHHs. The antibodies were found consistently and correctly by our tool.

**Figure 3.**
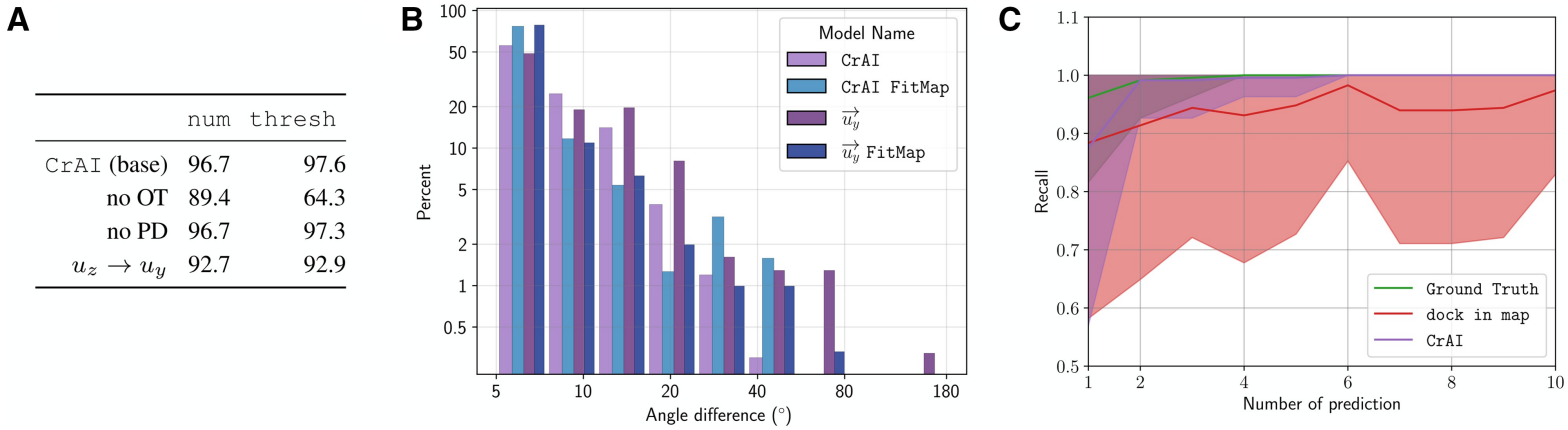
(A) F1 score of our model and ablations on the random Fab split. We try removing the optimal transport loss, the persistence diagram post-processing or training to predict $\vec{u_{y}}$ as a preferred axis instead of ${\vec{p}}_{A}$. (B) Distribution of angles of this model compared to our normal one. (C) F1 score of different approaches as a function of the number of predictions, in the VHH sorted setting. The solid line and shaded regions represent the mean and variance of the performance across systems.

*Ablation study.* To assess the relevance of different design choices of our approach, we retrain several models without specific individual features in our design. Since this means training a new model every time, we only perform this analysis in the Fab random split setting. We report the performance in the sys setting, but results are consistent in the ab setting. We try replacing Persistence Diagrams (PD) with a naive Non-Maximal Suppression (NMS) (Felzenszwalb *et al.* 2010, Girshick *et al.* 2014) that amounts to zeroing predictions around local minima. We also trained our model without using the Optimal Transport (OT) component, as is done in most classical object detection approaches (Girshick 2015, Redmon *et al.* 2016). We also tried to keep the method fixed, but to disrupt the template encoding by decomposing the rotation using ${\vec{u}}_{y}$, instead of ${\vec{u}}_{z}$, as the main vector. We present the results in Fig. 3A.

Persistence diagrams seem to enhance results in the thresh setting, with a limited impact. However, when removing optimal transport, performance collapses especially on the ability to predict the number of objects. This can be explained considering that antibodies cannot overlap (in contrast to detecting pedestrians in images for instance). Optimal transport helps to attribute a single detection to a region rather than enabling an arbitrary number of potentially overlapping/conflicting detections. Training the model with ${\vec{u}}_{y}$ also significantly weakens detection performance.

Finally, we show the histogram of angular error of the model trained using ${\vec{u}}_{y}$ in Fig. 3B. It has an angle error of respectively, $10.2^{{}^{\circ}}$ and $11.6^{{}^{\circ}}$, significantly higher on the vector prediction. Hence, predicting ${\vec{u}}_{z}$ is easier than ${\vec{u}}_{y}$, which validates the nonrestrictive inductive bias that we introduced in our formulation.

*Failure analysis.* Since the number of failed systems with CrAI is relatively low, we visually inspected *all of them*. Upon inspection, we found that approximately one third of errors were actually expected, because the deposited PDB only reported one asymmetrical unit of the map (inducing artifacts of false positives), or had manually placed antibodies in bad map regions.

Moreover, we noticed that most of our actual errors originate from the ordering and thresholding of the predictions and not from the detection of antibodies. To further validate this observation, we performed a study of the recall of CrAI and dock_in_map when forced to output a certain number of predictions. In Fig. 3C, we plot the fraction of VHHs captured by different approaches, when making a growing number of predictions per system. The green curve represents the best achievable results and does not always equal one as some systems contain multiple antibodies.

As can be seen in this figure, a small discrepancy exists between the ground truth and our tool. However, this discrepancy disappears around *k *=* *6 predictions, suggesting that with this number of detections per system our method captures *all* VHHs. dock_in_map has a much wider gap that tends to stay consistent despite allowing it to output more predictions. This further justifies that most of our errors originate from the thresholding. Thus, if our tool fails to detect an antibody, practitioners can ask for more predictions with a high chance of seeing it predicted. A more in-depth failure analysis, along with visualization of the dominant error modes, recall curves for other splits and extensive plotting of all failed systems can be seen in Supplementary Section S2.4.

## 4 Discussion

In this article, we addressed the problem of automatically finding the Abs in cryo-EM maps. This step currently constitutes a tedious, manual step thus hindering efficient and scalable structure estimation.

To achieve this goal, we proposed a customized solution, which exploits the structural properties and addresses the specific challenges of the problem, such as handling significant data scarcity and heterogeneity. Specifically, we leveraged the conserved structure of Fabs to cast this problem as an object detection one. We gathered and curated a database to enable a data-driven solution. Finally, we then designed a customized pose representation and loss based on optimal transport, which all help integrate prior information, while remaining efficient and flexible. Using our approach, no extra input is required to predict the number, position and pose of Fabs and VHHs in a map.

We validate our results on experimental maps and find the Ab positions with recall above 90%, which represents a 25% (resp 15%) improvement on Fabs (resp VHHs) over existing methods, while requiring no extra inputs and being *thousands of times faster* even when run on a single CPU. We show that the predicted pose correlates well with experiments, and illustrate our tool’s performance on six systems relevant to drug design.

We believe that this tool can reduce the burden on structural biologists working with cryo-EM maps of Abs and accelerate the resolution of 3D complexes of Abs bound to their antigen. In line with this objective, the method ships as a ChimeraX (Pettersen *et al.* 2004) bundle to enable seamless integration.

In the future, it will be interesting to see if our approach can be pretrained on X-ray maps in a similar manner to (Karolczak *et al.* 2024), expanded to other conserved families, beyond Fabs and VHHs, and enable placing several folded domains into a map, which has so far been done without machine learning (Liebschner *et al.* 2019, Chang *et al.* 2022, Wang *et al.* 2024). More broadly, we could replace a fixed template by a parametrized family of embeddings of the folded domains, expanding the expressive power of our framework.

The exceptional throughput of our tool opens the door to finding Abs in the output of heterogeneous reconstruction or even continuous distribution of maps, and thus to capture several modes of antibody binding. Moreover, the automatization of cryo-EM structure resolution by our technique enables the generation of antibody-antigen complexes at a larger scale, a critical step to improve our understanding and detailed modeling of antibody binding. More structural information will open possibilities for better and more accurate antibody modeling that will speed up the drug discovery process of bio-therapeutics.

## Supplementary Material

btaf157_Supplementary_Data

## Data Availability

The data underlying this article are publicly available in the PDB and SabDab databases. They can be processed, used to train and validate our model using our code, available in github.com/Vincentx15/crIA-EM, and archived in Zenodo with DOI 10.5281/zenodo.14967869.
