# Correction to: Finding antibodies in cryo-EM maps with CrAI

**DOI:** 10.1093/bioinformatics/btaf369

**Published:** 2025-06-30

This is a correction to: Vincent Mallet, Chiara Rapisarda, Hervé Minoux, Maks Ovsjanikov, Finding antibodies in cryo-EM maps with CrAI, *Bioinformatics*, Volume 41, Issue 5, May 2025, btaf157, https://doi.org/10.1093/bioinformatics/btaf157

In the version originally published, the third author’s affiliation was given as:

Integrated Drug Discovery, Structural Biology and Biophysics, Sanofi, Vitry-sur-Seine, 94400, France.

This has been updated to read:

Digital R&D, Sanofi, Vitry-sur-Seine, 94400, France.

