# Supplementary material for: Finding antibodies in cryo-EM maps with CrAI
Source: Bioinformatics. 2025 Apr 9;41(5):btaf157. doi: 10.1093/bioinformatics/btaf157 (PMC12077295; doi:10.1093/bioinformatics/btaf157)
Supplement: btaf157_Supplementary_Data [file btaf157_supplementary_data.pdf]

Appendix

In this Appendix, we first provide additional details on our methods by justifying the proposed decomposition of the rotations aligning a template into a query, listing modifications made to the SabDab database, detailing the neural network architecture used, providing a little primer on optimal transport and showing elements in favor of using a 10Å threshold.

In a second part, we provide additional results consisting of additional metrics regarding our detection performance, additional details on the validation on apo systems, a precise description of the case studies we chose to showcase our tool's applicability and an extensive analysis of the failure modes of our tool on its test set.

1 Methods

1.1 Proof of the validity of the decomposition

Following our notations, $R^*_{\mathbf{A}}$ is a rotation that aligns $\mathbf{A}$ onto $\mathbf{T}$. We want to show that $R^*_{\mathbf{A}}$ can be parametrized by the transformation of $p_{\mathbf{T}}$ into $p_{\mathbf{A}}$ along with a rotation around $p_{\mathbf{T}}$.

By construction :

$$\mathbf{A} = R^*_{\mathbf{A}}\mathbf{T} \Rightarrow p_{\mathbf{A}} = R^*_{\mathbf{A}} p_{\mathbf{T}}. \quad (1)$$

Let M be a rotation matrix of axis $p_{\mathbf{A}} \times p_{\mathbf{T}}$ and of angle ρ the angle between these vectors, so that it transforms one into the other. We can write :

$$p_{\mathbf{A}} = M p_{\mathbf{T}} \quad (2)$$

$$M^{-1} R^*_{\mathbf{A}} p_{\mathbf{T}} = p_{\mathbf{T}}. \quad (3)$$

Therefore, $M^{-1}R^*_{\mathbf{A}}$ is a rotation around the axis $p_{\mathbf{T}}$ by some angle θ, and we can write :

$$R^*_{\mathbf{A}} = M Rot_{p_{\mathbf{T}},\theta}. \quad (4)$$

1.2 Modifications to the SabDab database

We removed a few non canonical systems, such as fusion proteins or `8gq5` that contains 16 VHHs arranged in an unusual way and the obsolete `7ny5`. We then fixed edgy situations, often encountered when VHHs and Fabs are present in the system.

- When querying the Fabs, some chains were wrongly considered separate : 7YVN chain HI 7YVP chains IJ, 8HHX chains HI, 8HHY chains FW-GI, 8DXS chains FI-GJ, 7XOD chains RS-UV-XY, 7MLV chains KF, 6XJA (missing chain AB and missing antigen)
- VHHs were present when querying Fabs. They were annotated as having just one chain and no antigen partner : 7zlg-h-i-j chain K bound to L, 8hii-j-k chain N bound to L, 7xw6 chain N bound to AB, 7sk7 chain K bound to C, 7sk5 chain E bound to C, 7zyi chain K bound to L, 7xod chain T-W-Z bound to S-V-Y, 6ni2 chain A bound to B-V, 7wpe chain W-Z bound to V-Y, 6ww2 chain K bound to L, 7jhh chain N bound to L, 7ul3 chain C bound to A, 7tuy chain K bound to L
- Fabs were present when querying VHHs (often interacting with this VHH) : 7pij chain HL bound to N, 7wpd chain XY bound to A, 7wpf chain RS, UV, XY, 7wpf chain AC-HL bound to EN, 7php chain HL bound to N, 7m74 chain HL bound to A
- VHHs with no antigens were present in the VHH file (actually always bound to a Fab) : 7pij K bound to L, 7wpd Z bound to Y, 7nkr F bound to A, 7wpf T-W-Z bound to S-V-Y, 7wpf chain K-F bound to L-C, 7php-7m74-7jhg chain K, N, N bound to L, 7nis-7nk1-7nk2-7nk6-7nkc-7nj4-7nka chain F bound to A/B-A-A/B-A/B-B/C-A-A

1.3 Detailed architecture

We used a 3D Unet architecture. First let us define a `conv_block`(i,o) as three series of convolutions (with kernel size three and stride one), batch normalization and PreLu activations. The first convolution goes from i to o channels and the two others have o as a fixed number of channels.

In the encoding stage, we use a series of four `conv_blocks` of successive dimensions (1, 32, 64, 128, 256), alternated by a max pooling of kernel size 2 and stride 2.

In the decoding stage, we apply a transpose convolution (256, 256) with kernel size of 3 and stride 2 to the output of the encoder. We concatenate the result with the output from the third encoding `conv_block` to get 384 channels. We then run a `conv_block`(384, 128) on this concatenation.

Finally, we apply a last `conv_block`(128, 128) with only two series on the resulting tensor and replace the third one by a linear layer going from dimension 128 to dimension 10. The classification output dimensions are followed by sigmoid activation.

1.4 Optimal transport loss

After normalization, we can view $\hat{\mathcal{G}}$ and $\mathcal{G}$ as measures defined over the regular grid $\mathbf{g}$. The optimal transport cost between those measures is defined as :

$$\mathrm{OT}(\hat{\mathcal{G}}, \mathcal{G}) \overset{\text{def.}}{=} \min_{\pi \in \Pi_{\hat{\mathcal{G}},\mathcal{G}}} \int_{\mathbf{g}^2} \|x-y\|^2 d\pi(x,y).$$

Where $\Pi_{\hat{\mathcal{G}},\mathcal{G}}$ is the set of coupling measures on $\mathbf{g}$ with marginals $\hat{\mathcal{G}}$ and $\mathcal{G}$. The actual computation of this term is prohibitively expensive, hence we add a regularization term with weight $\varepsilon > 0$ and introduce :

$$\mathrm{OT}_\varepsilon(\hat{\mathcal{G}}, \mathcal{G}) \overset{\text{def.}}{=} \min_{\pi \in \Pi_{\hat{\mathcal{G}},\mathcal{G}}} \int_{\mathcal{X}^2} \|x-y\|^2 d\pi(x,y) + \varepsilon \mathrm{KL}(\pi \mid \hat{\mathcal{G}} \otimes \mathcal{G})$$

$$\text{where } \mathrm{KL}(\pi \mid \hat{\mathcal{G}} \otimes \mathcal{G}) \overset{\text{def.}}{=} \int_{\mathcal{X}^2} \log\left(\frac{\mathrm{d}\pi}{\mathrm{d}\hat{\mathcal{G}}\mathrm{d}\mathcal{G}}\right) \mathrm{d}\pi.$$

We notice that $\mathrm{OT}_0 = \mathrm{OT}$ and for any $\varepsilon > 0$, adding this εKL regularization makes the computation tractable. However, this regularization term also introduces the so-called *entropic bias* since for a measure x, $\mathrm{OT}_\varepsilon(x, x) > 0$. Sinkhorn divergences were introduced to correct for this bias while remaining tractable, and that is what we use as a loss :

$$\mathcal{L}_2(\hat{\mathcal{G}}, \mathcal{G}) = \mathrm{OT}_\varepsilon(\hat{\mathcal{G}}, \mathcal{G}) - \frac{1}{2}\mathrm{OT}_\varepsilon(\hat{\mathcal{G}}, \hat{\mathcal{G}}) - \frac{1}{2}\mathrm{OT}_\varepsilon(\mathcal{G}, \mathcal{G})$$

1.5 Finding the optimal threshold

We present the error in the number of Abs detected for different thresholds in Figure A.1. Throughout datasets, persistence diagrams seem to give a better estimate. The overall best value is achieved for a threshold of approximately 0.2 and is not very sensitive to this threshold.

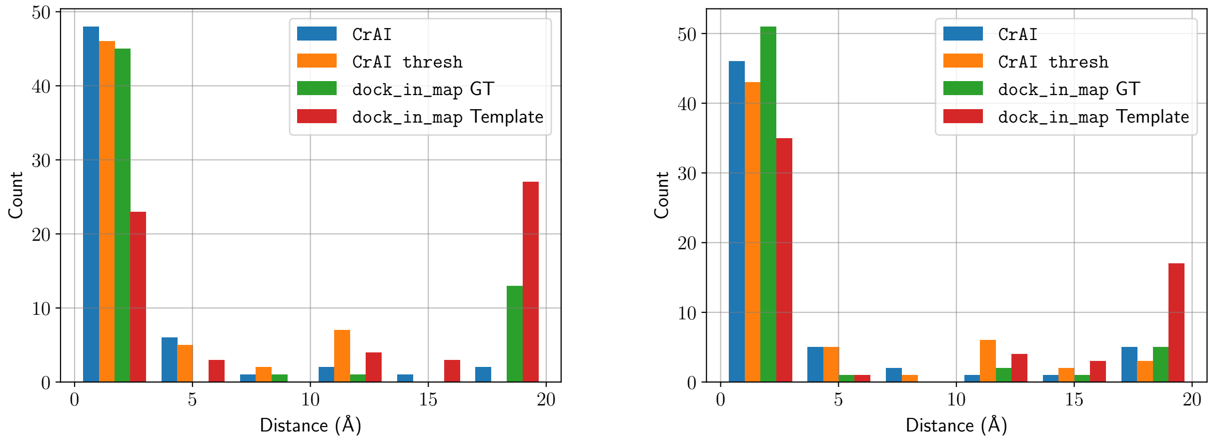

Fig. A.2: Capped histogram of distances between predictions and experimental structures on Fabs. `random` is on the left and `sorted` on the right.

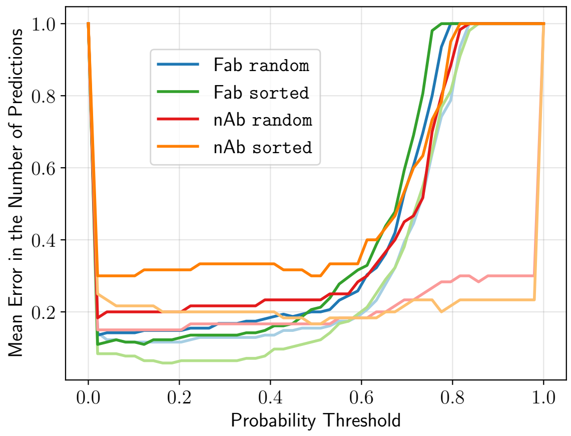

Fig. A.1: Finding the best threshold for selecting the right number of systems. x-axis represents the threshold value. y-axis represents the mean absolute error in the number of predicted systems over the data set. Different colors represent different data sets. Darker lines represent a naïve approach for NMS while the lighter ones are computed using persistence diagrams.

2 Results

2.1 Fab detection performance

We compute the distances from prediction to ground truth obtained from our tool and `dock_in_map`. For a nicer visualization, we cap all distances to 20Å. We also count failed systems and errors as 20Å predictions so that the bar at 20Å represents all failures of a tool. We show the result in Figure A.2

As can be seen, our tool widely outperforms `dock_in_map`. Under the hit-rate metric, we have the following performances (by order of tools) : `random` : 94.1, 88.8, 62.2, 19.4 `sorted` : 97.3, 93.9, 71.1, 8.5.

Based on this histogram analysis, we define a true positive as a prediction whose center of mass is closer than 10Å to the ground truth and usually report performance with the F1 score that is the harmonic mean of precision and recall. In settings where the number of objects is a given, each false negative (missed prediction) generates a false positive (extra prediction) and hence precision, recall and F1 score are all equal. We report the value of those metrics in the *thresh* setting in Supplementary Table A.1.

Performance on Fabs	Metric	`random` Split `sys`	`ab`	`sorted` Split `sys`	`ab`	Mean `sys`	`ab`
`dock_in_map`	F1	66.6	61.9	71.3	66.0	69.0	64.0
`CrAI num`	F1	97.3	96.7	**96.9**	**95.7**	**97.1**	96.2
`CrAI thresh`	F1	**98.1**	**97.6**	96.1	**95.7**	**97.1**	**96.7**
`CrAI thresh`	Precision	98.0	96.7	97.6	97.0	97.4	97.2
`CrAI thresh`	Recall	99.0	98.5	96.6	94.4	98.8	97.6
Performance on VHHs		F1 (`sys`)	F1 (`ab`)	F1 (`sys`)	F1 (`ab`)	F1 (`sys`)	F1 (`ab`)
`dock_in_map`	F1	77.8	68.8	90.5	89.1	84.2	79.0
`CrAI num`	F1	**100.0**	**100.0**	**90.9**	**90.6**	**95.5**	**95.3**
`CrAI thresh`	F1	99.4	99.3	90.3	88.4	94.9	93.9
`CrAI thresh`	Precision	100.0	100.0	90.5	87.7	100.0	95.3
`CrAI thresh`	Recall	99.1	98.7	92.7	89.1	98.9	95.7

Table A.1. Precision and recall values for the detection performance assessed on `CrAI thresh`. As explained in the text, in the `num` setting and for `dock_in_map`, those values are equal, and equal to the F1 score since the number of false positive and false negative are the same.

2.2 Validation on apo systems

The list of systems found by fetching UniProt codes present in our database that were not included in SabDab and were not viruses is the following: 7Y1Q, 8HK5, 8U4N, 7SYF, 8F7S, 7YDQ, 7TX6, 8U8F, 8C03, 8DMI, 8DGS, 7PVD, 9F33, 7Y1R, 3J0G, 8JRU, 8DNG, 7ANW, 8IWP, 6DZY, 7ZJ6, 8D74, 8SAT, 7L89, 7PHR, 7RU2.

We fetched all corresponding cryo-EM maps and performed an uncropped prediction on those maps. Among those systems, 12 are predicted to include an antibody. Upon manual inspection, we found that 8 actually contained antibodies, but were missing in the SabDab database: 8C03, 9F33, 8JRU, 8HK5, 8U4N, 6DZY, 8SAT, 7L89. We find it interesting that two thirds of our errors are actually labeling errors in the SabDab database.

Among the four remaining systems, two of these false predictions disappeared when using a custom threshold (8F7S, 7RU2) and two persisted as fake predictions (7ANW, 8IWP).

2.3 Case study

Detailed evaluation results for 6 systems of the random split test set are shown in Figure A.3. Those six systems were picked because of their relevance in the context of drug discovery, at resolutions ranging from 3 to 8Å, and include four Fabs whose location was found by our tool consistently and correctly. `dock_in_map` on the other hand, only finds the right location in one case, predicts a shifted location on another and completely fails its prediction on the last ones. The two remaining systems, with resolutions of 3.1 and 3.8Å respectively, contain VHHs for which `CrAI` detects the location correctly and for which `dock_in_map` fails. There were no examples in which dock in map succeeded and `CrAI` failed. Of the 6 systems shown, 4 used Chimera for manual docking, one doesn't mention the docking program and one uses MDFF (Trabuco *et al.*, 2009), a molecular dynamics software not commonly employed by experimental structural biologists. It is hard to evaluate ChimeraX placement in the map as the time and the success of placement isn't automatic and it is user dependent. For the lower resolution structures, chain building programs are not suitable as resolutions better than 3.5 Å are needed.

The first two examples are low resolution illustrations of the structural characterization of neutralizing antibodies, in the context of cervical cancer for the Human papillomavirus type 58 pseudovirus (HPV58) in complex with the Fab fragment of 5G9 at a resolution of 6.41Å (He *et al.*, 2021)(Figure 3a) and the viral infection with tetanus for the neurotoxin LC-HN domain in complex with TT110- Fab1 at a resolution of 8.00Å (Pirazzini *et al.*, 2021) (Figure 3b). While the low resolution of the first example does not preclude both `CrAI` and `dock_in_map` to correctly predict the position of the Fab, at 8.0 Å, only `CrAI` correctly positions the Fab. Membrane proteins are notoriously difficult to characterize structurally and are often solved at lower resolutions than soluble ones. Structural information is important to decipher the signaling and regulatory mechanisms of this class of proteins. G protein-coupled receptors (GPCRs) in Figure 3c and d play a crucial role in signaling and are one of the major target families of drugs. Three Fabs are present in the density of beta-arrestin2 (Figure 3c) in complex with a phosphopeptide corresponding to the human C5a anaphylatoxin chemotactic receptor 1 C5aR1 at a resolution of 4.40Å (Maharana *et al.*, 2023). `CrAI` correctly finds all of them while `dock_in_map` misses to correctly predict two out of three. In Figure 3d, the class A orphan GPCR (GPR139), (Zhou *et al.*, 2021) is captured with and stabilized by Nb35 in different conformational states highlighting allosteric modulation roles of VHHs in GPCRs. In this first example with a VHH in a protein complex, `CrAI` correctly finds the location of the VHH while `dock_in_map` fails. In the fight against covid-19, a search for potent and broad-spectrum coronavirus blockers was initiated and many antibodies were shown to block the entry of several variants of SARS-CoV-2 (SARS-CoV-2, SARS-CoV-2-D614G, B.1.1.7, B.1.351, B.1.617.1, P.1, SARS-CoV, and HCoV-NL63) by binding to human ACE2, without causing severe side effects. In Figure 3e, ACE2 is found in complex with targeting monoclonal antibodies at a resolution of 3.2Å (Chen *et al.*, 2021). This system illustrates `CrAI` correctly finds the two Fabs in the density, while `dock_in_map` is unsuccessful. The final example in Figure 3f is the Cryo-EM structure of SPCA1a in E1-Ca-AMPPCP state subclass 3 at a resolution 3.14Å (Chen *et al.*, 2023). In this case, with a medium resolution, the location of the VHH Nb14 is properly found by `CrAI`, however, its orientation is not correct. When analyzing this protein complex, it appears that the binding mode of Nb14 to its antigen, the Calcium transporting ATPase type 2C member 1, is non-conventional, with the framework region of Nb14 lying on the surface of the antigen while the CDRs are not oriented toward the epitope. The prediction made by `CrAI` followed the conventional binding mode of a VHH, with the CDRs facing the epitope of the antigen. This specific example highlights a potential bias of `CrAI` in correctly predicting the orientation and the binding mode of antibodies and VHH. It can be noted that in this specific case, `dock_in_map` fails to identify the correct location of Nb14.

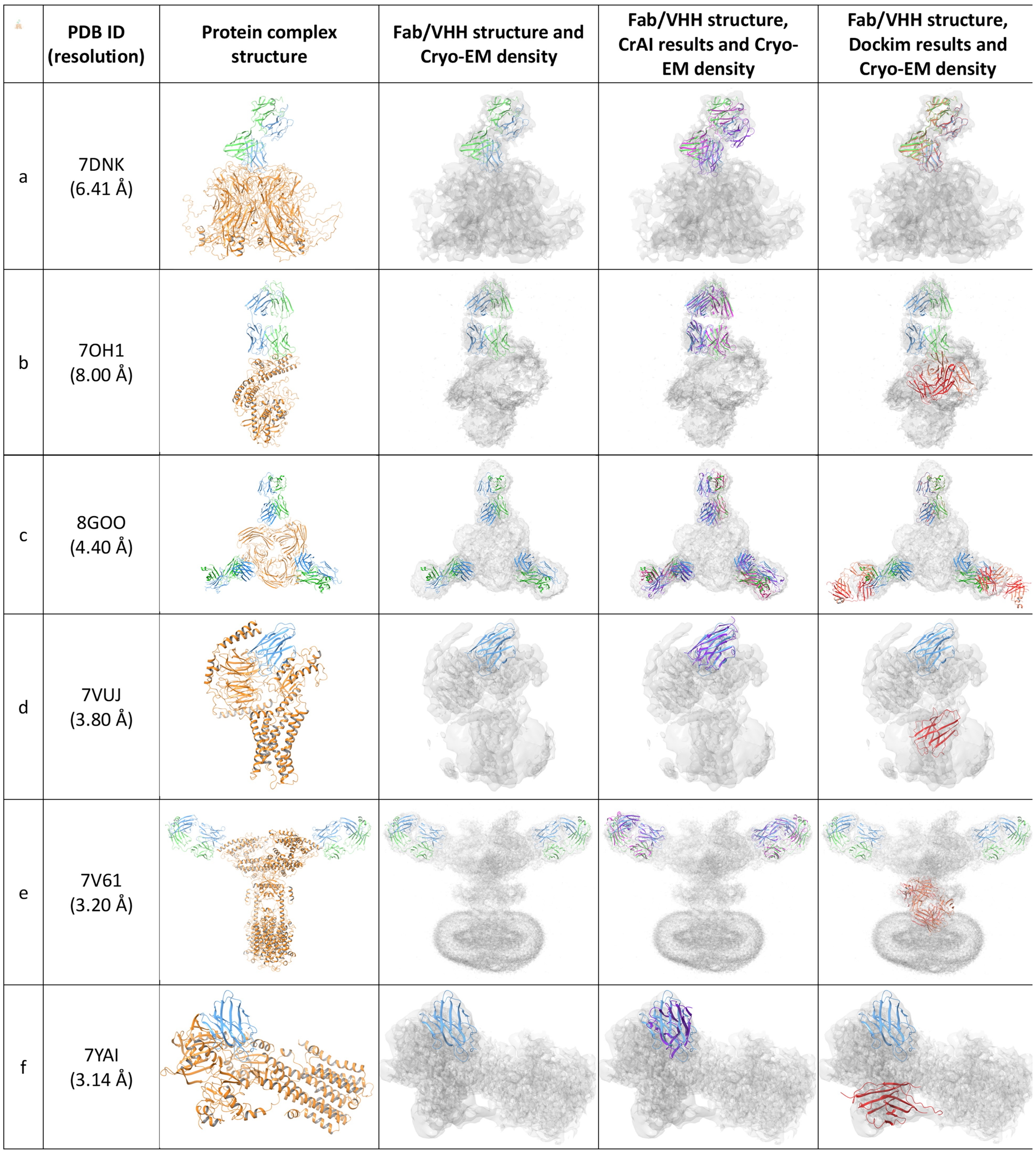

Fig. A.3: In this Table, columns correspond to 1) PDB code and EM resolution; 2) Protein structures from the PDB (the antigen is displayed in orange, the heavy chain in blue and the light chain in green); 3) EM map and corresponding structure; 4) Superimposition of the Fab or the VHH from the PDB structure (blue/green) with the `CrAI` results (purple); 5) Superimposition of the Fab or the VHH from the PDB structure with the `dock_in_map` results (red).

The rows correspond to six systems of our test set: (a) Human papillomavirus type 58 pseudovirus (HPV58) in complex with the Fab fragment of 5G9 at a resolution of 6.41Å, (b) Tetanus neurotoxin LC-HN domain in complex with TT110- Fab1 at a resolution of 8.00Å, (c) Structure of beta-arrestin2 in complex with a phosphopeptide corresponding to the human C5a anaphylatoxin chemotactic receptor 1 C5aR1 at a resolution of 4.40Å, (d) Cryo-EM structure of a protein complex containing a class A orphan GPCR (GPR139), stabilized by a VHH (Nb35) at a resolution of 3.80Å, (e) ACE2 -Targeting Monoclonal Antibody as Potent and Broad-Spectrum Coronavirus Blocker at a resolution of 3.2Å, (f) Cryo-EM structure of SPCA1a in E1-Ca-AMPPCP state subclass 3 at a resolution 3.14Å.

Error type	Count sys	Count ab
Extra Abs	13	14
- correctly placed	*5*	*6*
Missing Abs	14	26
- bad density	*7*	*9*
Others	2	2

Table A.2: A complete classification of all errors by `CrAI` on the `sorted` split for both Fabs and VHHs.

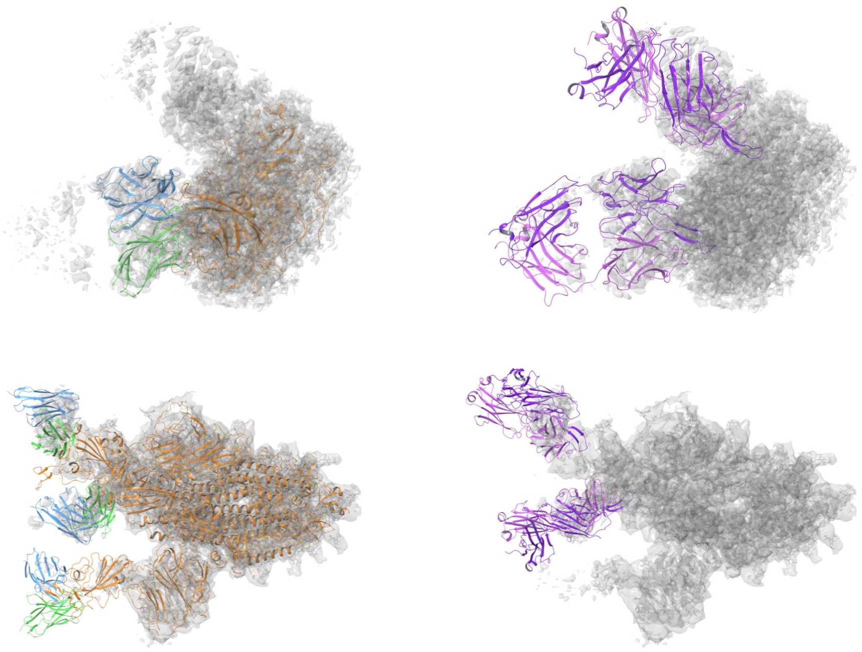

Fig. A.4: Illustration of the two main modes of failures in two examples. The first row corresponds to `8E8R` and the second to `8HEC`. Most errors result from predicting more abs - including scenarios where these extra predictions are reasonable like for `8E8R` - or fewer abs - including scenarios where the missing ab falls in a region with very poor density like in `8HEC`.

2.4 Failure analysis

Since the number of failed systems with `CrAI` is relatively low, we visualized all *all of them* (for the `sorted` split) in Figure A.6. After inspecting these failures, we identified two dominant sources of errors as reported in Table A.2. A visual depiction of these two error categories is also shown in Figure A.4.

The vast majority of failures amount to either predicting too many antibodies or not predicting enough.

When predicting too many Abs, interestingly, the right ones are *always* included in our prediction. Moreover, in 5 out of these 15 errors, the extra predictions were found to correspond to an antibody that *actually appears in the density*, such as an element from another asymmetrical unit, and thus can be considered as accurate detections. In the eight remaining cases, two extra predictions result from a wrong ordering (the wrong prediction is ranked first) and four result from a faulty thresholding: the artifacts have lower probabilities but fail to be automatically discarded. Overall, we remark that these additional predictions are both infrequent and can easily be discarded by practitioners.

Furthermore, predictions for 14 systems lack one or more Ab. In over a third of the cases, this could be explained by a poor density of the map around the missing antibody. Forcing `CrAI` to produce additional predictions on those 12 systems, we observe that 16 out of 20 missing Abs can be found, often after just one or two extra predictions. Thus, our software offers the option to predict a certain *number* of predictions instead of relying on automatic thresholding, to allow practitioners to capture potential missing annotations.

In summary, we noticed that most of our errors originate from the ordering and thresholding of the predictions and not from the detection of antibodies.

To further validate this observation, we performed a study of the accuracy of `CrAI` and `dock_in_map` across a varying number of predictions. For this, we forced both methods to output up to ten predictions and then computed the recalls obtained when keeping the k-best predictions. While our method can easily output ten predictions, to get this result for `dock_in_map` we independently ran it on a growing set of inputs. The first few inputs correspond to the observed structures of antibodies while additional ones are copies of these structures in a random order.

In Figure A.5 we plot the fraction of all antibodies captured by different approaches, when making a growing number of predictions per system. The blue curve represents the best achievable results. It does not always equal one as some systems contain multiple antibodies. As can be seen in this figure, a small discrepancy exists between the ground truth and our tool. However this discrepancy disappears around $k = 6$ predictions, suggesting that with this number of detections per system our method captures *all* antibodies. This further justifies that most of our errors originate from the thresholding. Thus, if our tool fails to detect an antibody, practitioners can ask for more predictions with a high chance of seeing it predicted. `dock_in_map` has a much wider gap that tends to stay consistent despite allowing it to output more predictions.

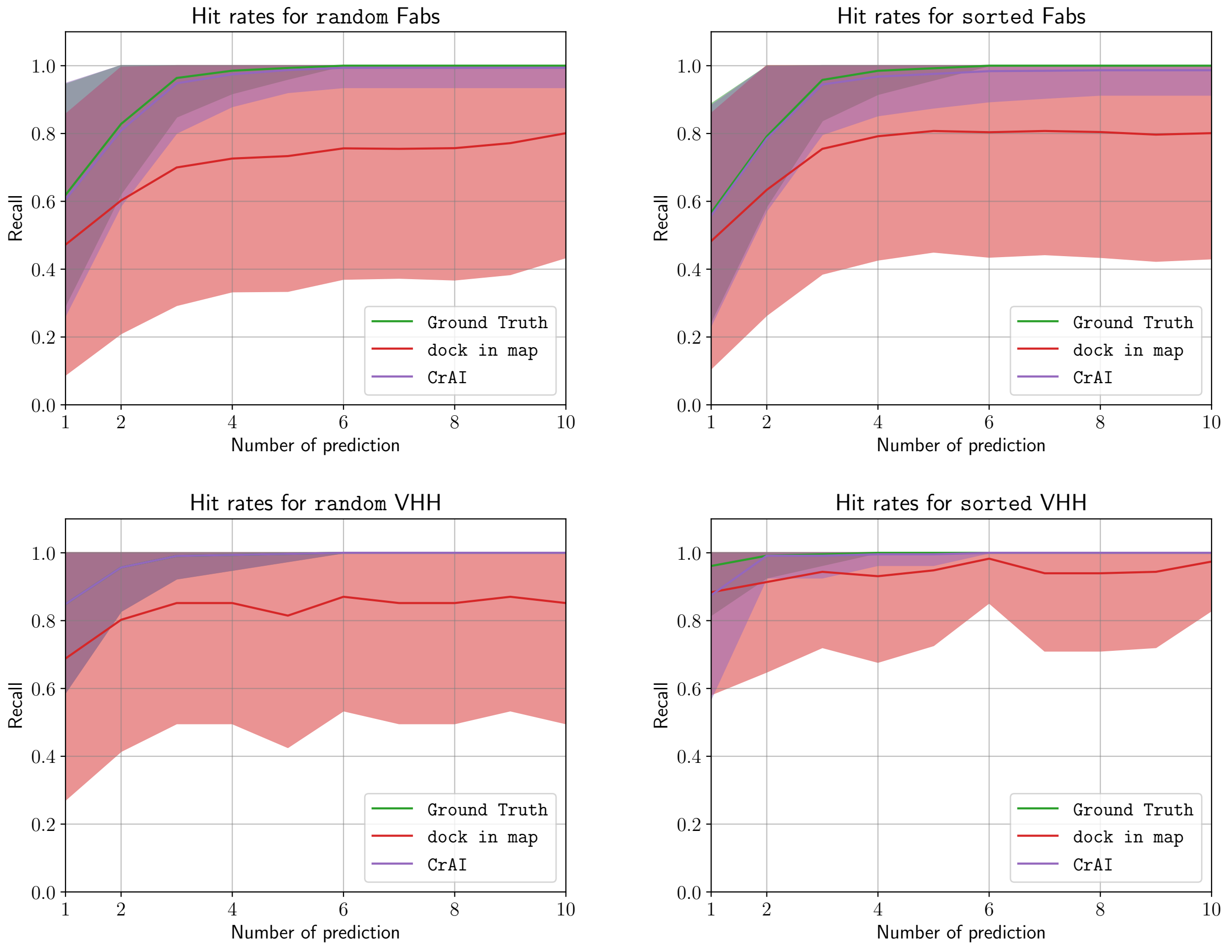

Fig. A.5: Recalls of different approaches (y axis) as a function of the number of predictions (x axis) in our four data settings. The solid lines represent the mean performance of systems and shaded regions correspond to the variance. Even if we observe a small discrepancy between our prediction and the ground truth line, note that most errors originate from the automatic thresholding feature, and our method achieves near perfect recall with as few as 6 detections, compared to `dock_in_map`, which fails to capture a significant number of present structures.

Predicted PDB	Ground truth	PDB File & Details of the error
**Metrics computation was wrong**		
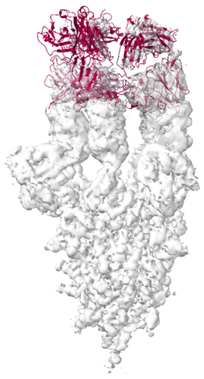	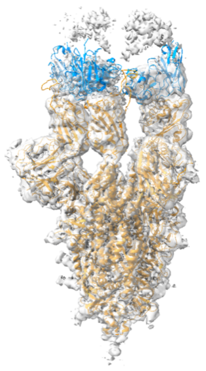	`7YVN`<br>Not an error
**Too many objects**		
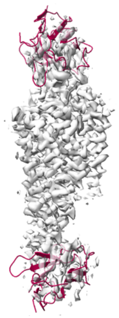	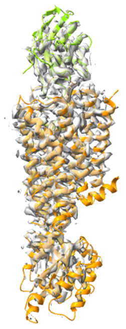	`7YM8`<br>1 extra VHH fit in a helical domain<br><br>=> extra ranked 2
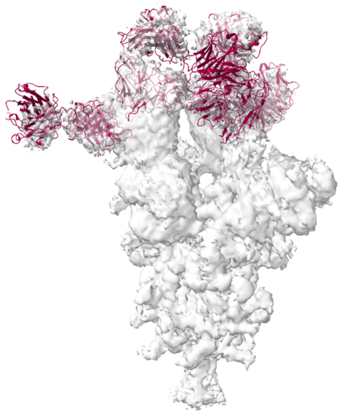	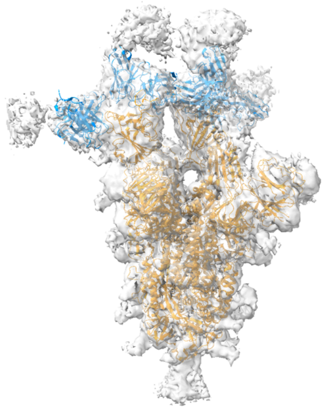	`7YVP`<br>1 extra Fab<br>Not a clear density for the extra Fab<br><br>=> extra ranked 4 (last)

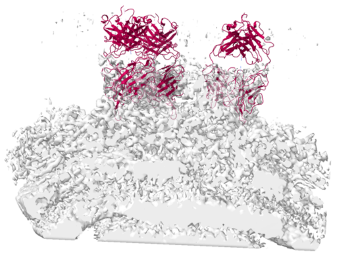	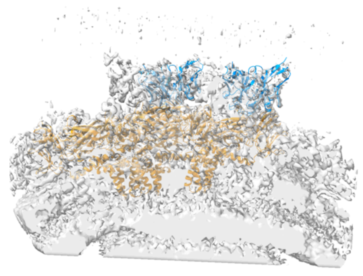	8CXI<br>1 extra placed correctly
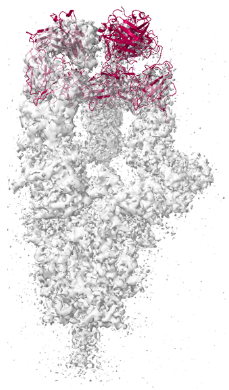	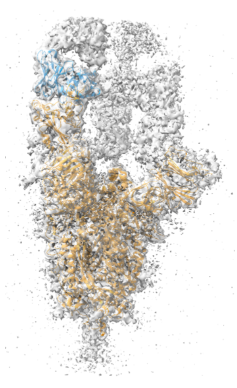	8D0Z<br>2 extra placed correctly
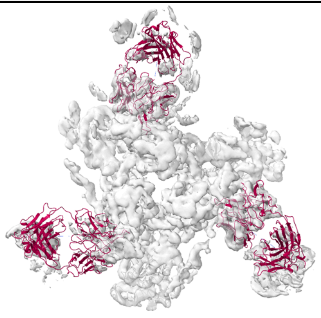	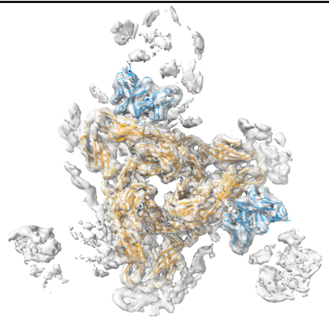	8DEF<br>1 extra placed correctly for which the density is weak.
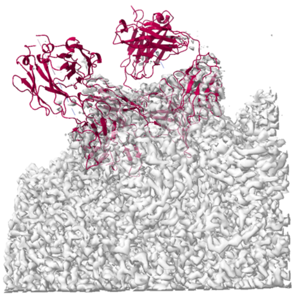	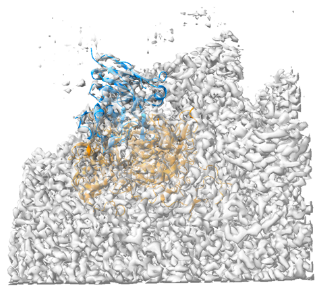	8E8R<br>1 extra placed correctly

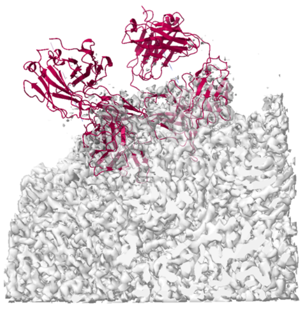	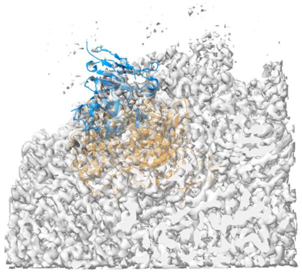	8E8X<br>1 extra placed correctly
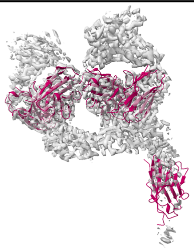	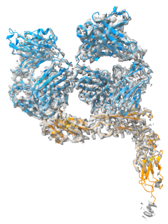	8D7E<br>1 extra VHH<br><br>-> ranked 3rd
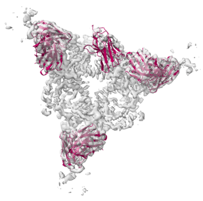	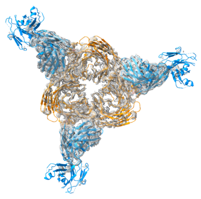	8GOC<br>1 extra VHH<br><br>-> ranked 4th
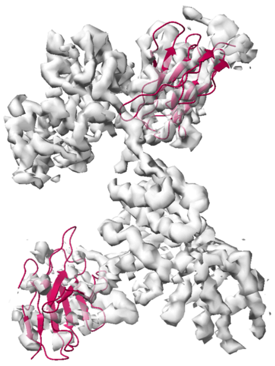	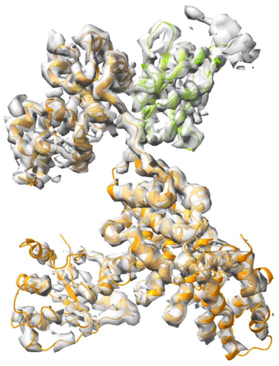	8GNJ<br>1 extra VHH<br><br>=> ranked 0th

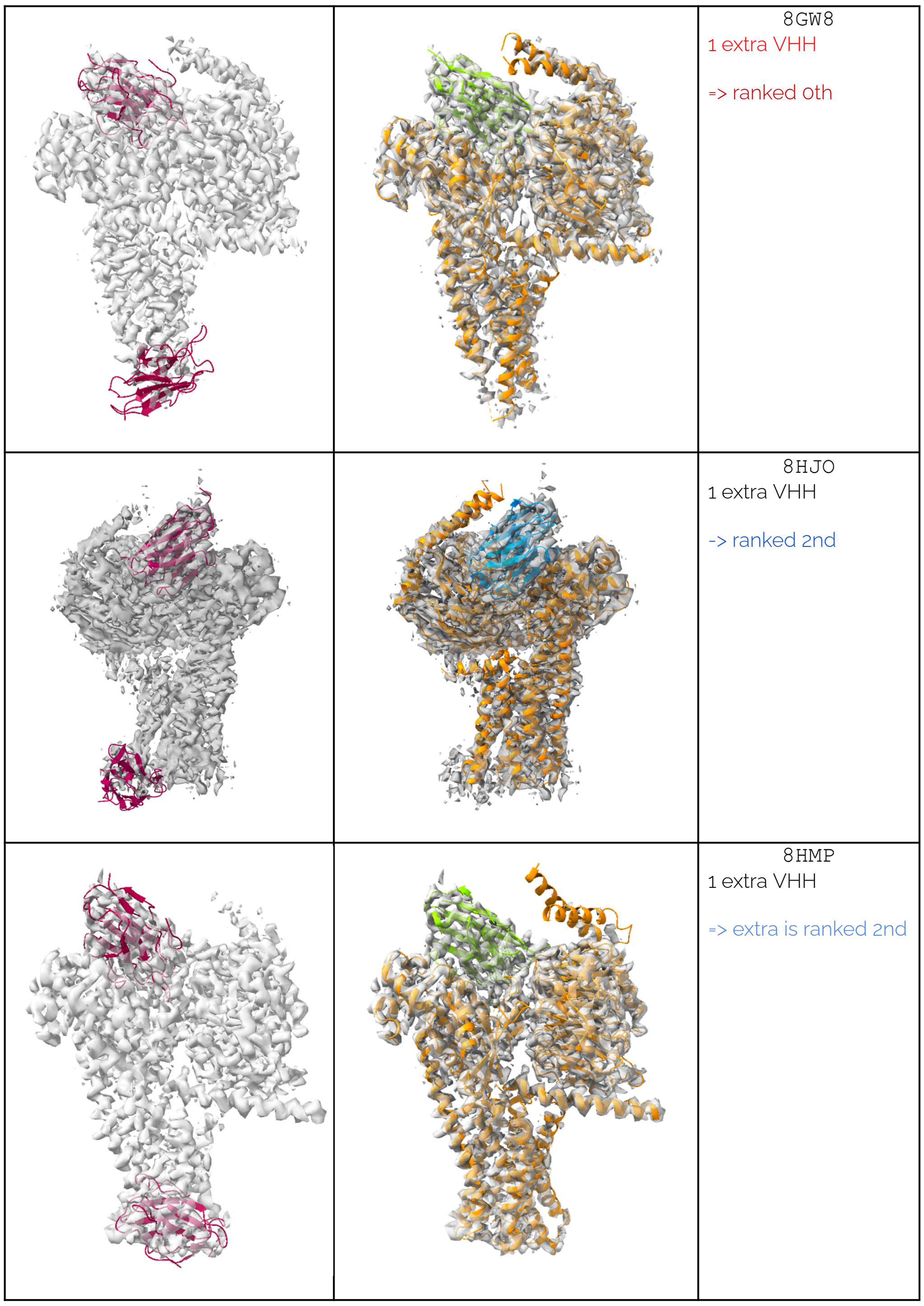
8GW8
1 extra VHH
=> ranked 0th
8HJO
1 extra VHH
-> ranked 2nd
8HMP
1 extra VHH
=> extra is ranked 2nd

Missing objects		
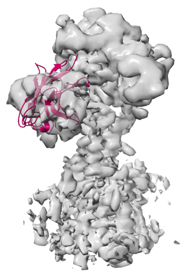	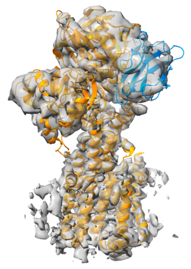	`7YAJ`<br>1 less<br>1 nanobody badly placed<br><br>Bad density 1
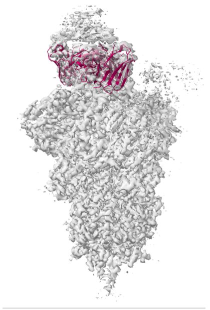	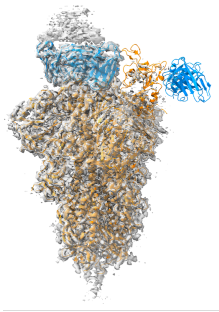	`7XDB`<br>1 less with no density<br><br>Bad density 1
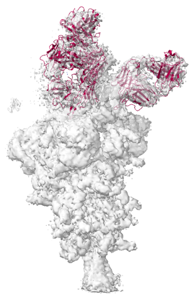	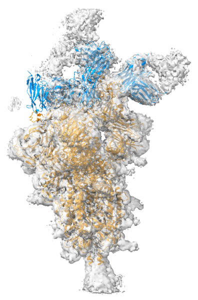	`7XJ6`<br>1 less with bad density<br><br>Bad density 1<br><br>=> ranked 5th
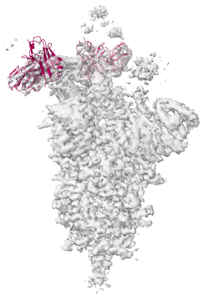	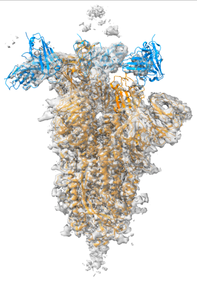	`7YVI`<br>1 less

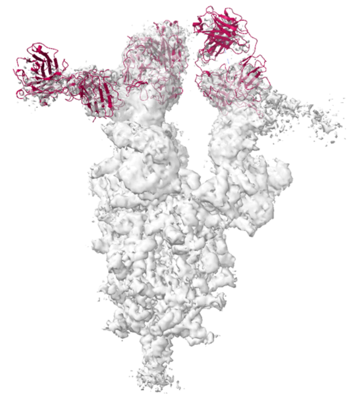	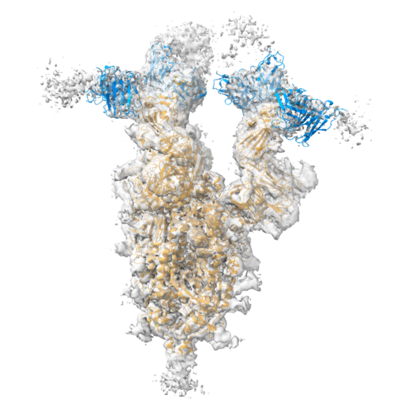	`7YVO`<br>1 less with bad density<br><br>Bad density 1 for the 4th Fab
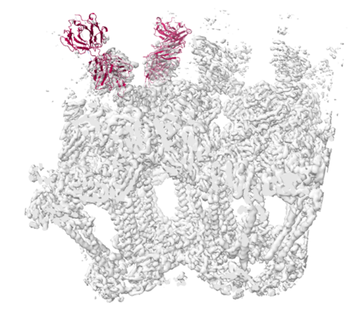	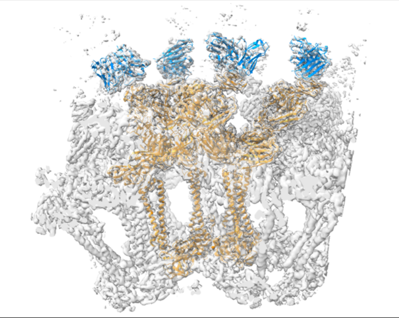	`8DWW`<br>3 less<br><br>=> ranked 6, missed
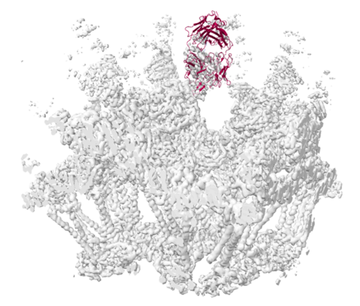	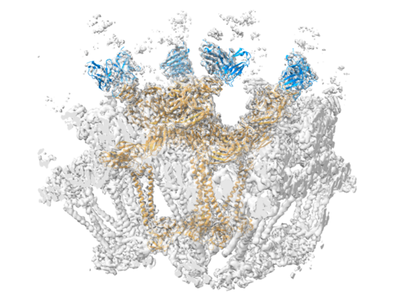	`8DWX`<br>3 less<br><br>=> ranked 2,4,5
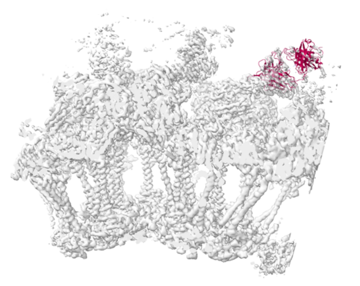	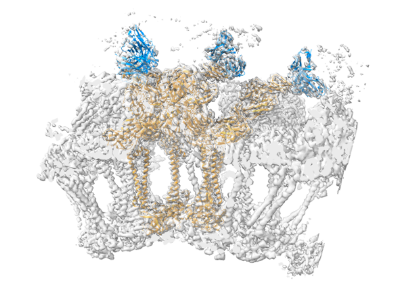	`8DWY`<br>3 less<br><br>=> ranked 2,8,missed

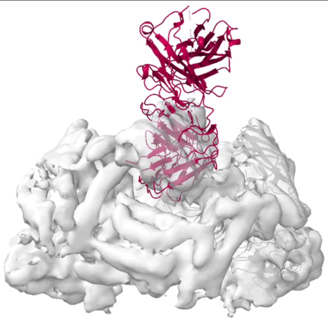	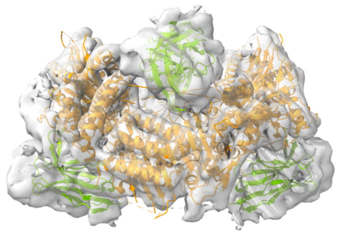	`8EQB`<br>3 less<br>Fab in place of a nanobody<br><br>=> ranked 2,4,6
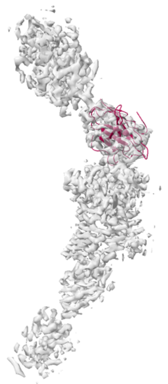	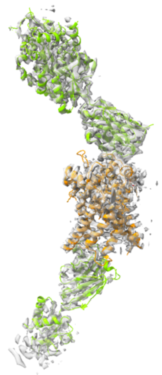	`8G8W`<br>1 less<br>Atypic system (megabodies)<br><br>=> ranked 2
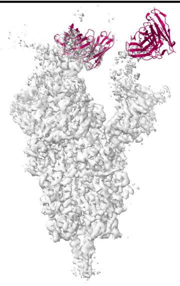	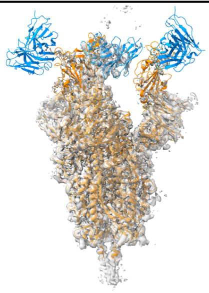	`8GTP`<br>1 less with weak density<br><br>bad density 1<br><br>-> ranked 3rd

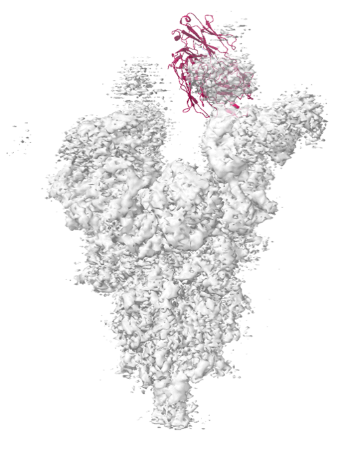	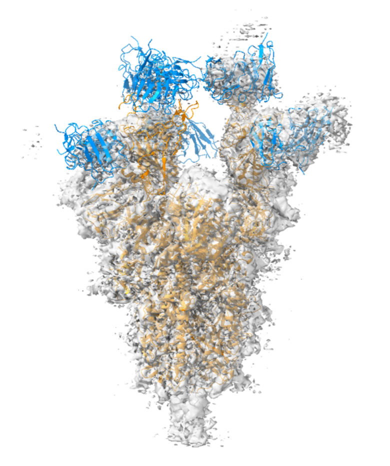	`8H07`<br>5 less including 3 with bad density<br><br>bad density 3
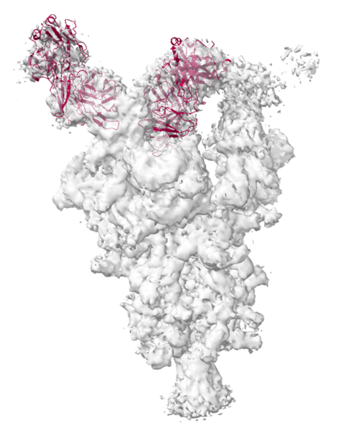	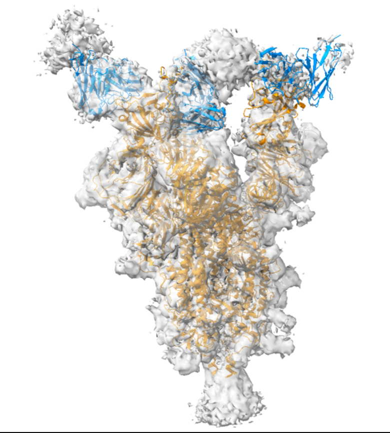	`8HEC`<br>1 less with bad density<br><br>=> ranked 4th<br><br>bad density 1
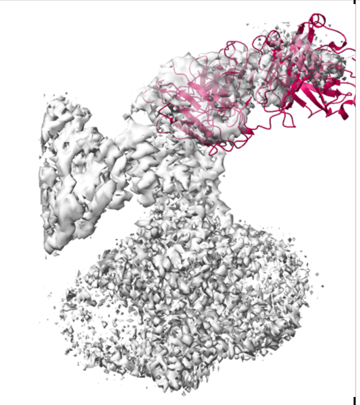	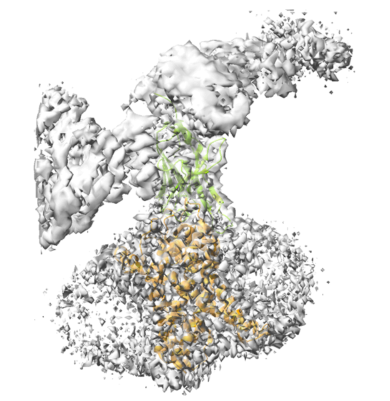	`8J1N`<br>1 less<br><br>1 more Fab (but there is a density that seems like a Fab)<br><br>=> ranked 2nd

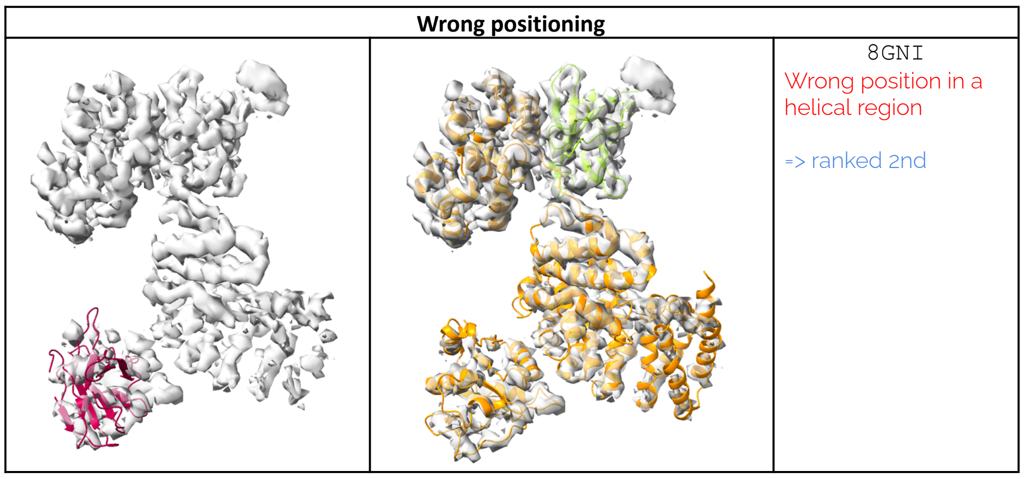

Fig. A.6: All failures, split by error type
